# Clinical Impact of a LAG3 Single-Nucleotide Polymorphism in Relapsed, Refractory DLBCL Patients Treated with Glofitamab

**DOI:** 10.3390/cancers18060930

**Published:** 2026-03-13

**Authors:** Maeva Ullmann, Katja Seipel, Henning Nilius, Martina Bertschinger, Vera Rentsch, Ulrike Bacher, Thomas Pabst

**Affiliations:** 1 Department of Medical Oncology, Inselspital, University of Bern, 3010 Bern, Switzerland; maeva.ullmann@students.unibe.ch (M.U.); martina.bertschinger@insel.ch (M.B.); vera.rentsch@spitalstsag.ch (V.R.); 2 Department for Biomedical Research (DBMR), University of Bern, 3008 Bern, Switzerland; veraulrike.bacher@insel.ch; 3 Department of Clinical Chemistry, Inselspital, Bern University Hospital, 3010 Bern, Switzerland; henning.nilius@insel.ch; 4 Department of Hematology, Inselspital, University of Bern, 3010 Bern, Switzerland

**Keywords:** diffuse large B-cell lymphoma (DLBCL), glofitamab, lymphocyte-activation gene 3 (LAG3), T-lymphocyte-associated protein 4 (CTLA4), single nucleotide polymorphism (SNP), bispecific antibody (BsAb), bispecific T-cell engager (BiTE)

## Abstract

Background: Glofitamab is a bispecific antibody engaging CD3 on T-cells and CD20 on B-cells. Glofitamab is approved for the treatment of relapsed, refractory diffuse large B-cell lymphoma (R/R DLBCL). Lymphocyte-activation gene 3 (LAG3) and T-lymphocyte-associated protein 4 (CTLA4) are immune checkpoint receptors with inhibitory effects on T-cell activity. There are several common germline variants of both receptor genes. Methods: Here, we evaluate clinical outcomes in R/R DLBCL patients treated with glofitamab according to the single-nucleotide polymorphisms *LAG3* rs870849 and *CTLA4* rs231775. Results: While there was no apparent association of *CTLA4* genotype with glofitamab treatment outcomes, significant differences emerged in *LAG3* rs870849 carriers with extended progression-free and overall survival in homozygous *LAG3* T455, intermediate PFS and OS in heterozygous *LAG3* I455T, and short PFS and OS in homozygous *LAG3* I455 carriers. Conclusions: *LAG3* rs870849 may be a prognostic response marker in R/R DLBCL treated with glofitamab.

## Simple Summary

Glofitamab is a newly approved bispecific antibody for the treatment of relapsed, refractory diffuse large B-cell lymphoma. Treatment response may vary depending on the genetic background of the patient. Here, we evaluate clinical outcomes in patients with DLBCL treated with glofitamab according to *LAG3* and *CTLA4* genotypes. While there was no apparent clinical impact associated with the *CTLA4* genotype, there was a significant association of the *LAG3* genotype with clinical response. Future personalized therapeutic strategies may evolve in consideration of a specific *LAG3* gene polymorphism.

## 1. Introduction

Diffuse large B-cell lymphoma (DLBCL) is the most frequent aggressive non-Hodgkin lymphoma [1,2,3,4]. While around 60% of DLBCL patients can be cured by frontline therapy with R-CHOP, a significant proportion of patients experience relapse or refractory disease [1,5,6]. Glofitamab is a bispecific antibody (BsAb) and T-cell engager (BiTE) binding CD3 on endogenous T-cells and CD20 on malignant B-cells, resulting in T-cell activation and tumor cell lysis [5,7,8]. Glofitamab has emerged as an effective immunotherapy for relapsed or refractory DLBCL (R/R DLBCL), with durable response in heavily pretreated patients [5,9,10]. Glofitamab therapy is approved for R/R DLBCL after two or more prior lines of systemic therapy [11]. There is marked variability in glofitamab response rates among R/R DLBCL patients, indicating that patients’ immune systems may affect clinical outcomes.

The efficacy of glofitamab therapy depends on the presence of functional T-cells [7,12]. Lymphocyte Activation Gene 3 (LAG3) and T-lymphocyte-associated protein 4 (CTLA4) are immune checkpoint receptors that are both expressed on T-cells [13,14]. Common germline single-nucleotide polymorph (SNP) variants in *CTLA4* and *LAG3* genes have been associated with clinical response in DLBCL treated with CAR T-cell therapy [15,16]. By transmitting inhibitory signals, LAG3 and CTLA4 can limit T-cell expansion and inhibit effector function [17,18]. *LAG3* expression is significantly upregulated in DLBCL [19,20,21]. Elevated LAG3 expression and specific *LAG3* gene polymorphisms are associated with an impaired T-cell response and inferior outcomes in patients treated with T-cell-engaging therapies, such as CAR T-cell therapy in DLBCL [16]. Preclinical studies indicate that T-cell exhaustion, characterized by upregulation of inhibitory receptors such as LAG3, can reduce the cytotoxic potential of endogenous T-cells, which are engaged by glofitamab [7,22].

Combining glofitamab with an immune checkpoint blockade by LAG3 inhibition can increase T-cell activation, maintain effector function, and improve antitumor efficacy in lymphoma models [22]. This supports the hypothesis that LAG3-mediated immune regulation is an important determinant in the response to glofitamab. Moreover, the *LAG3* genotype may serve as predictive biomarker for the response to BsAb therapy in DLBCL [9,22,23]. In this exploratory investigation, we evaluated clinical outcomes in R/R DLBCL patients treated with glofitamab according to *LAG3* rs870849 and *CTLA4* rs231775 SNPs.

## 2. Materials and Methods

### 2.1. Patients

This retrospective, single-center observational study included 28 DLBCL patients treated with glofitamab in 2020–2025 at the University Hospital of Bern, Switzerland.

As per established standard-of-care procedure [6], patients received one single dose of 1000 mg obinutuzumab (Gazyvaro^®^, Roche, Basel, Switzerland) on cycle 1 day 1 (C1D1) to achieve a depletion of B-cells in peripheral blood and secondary lymphoid tissues, thereby attenuating subsequent T-cell activation and reducing the incidence and severity of cytokine release syndrome (CRS). Thereafter, step-up dosing of glofitamab was performed with 2.5 mg on C1D8 and 10 mg on C1D15, followed by administration of a fixed 30 mg dose every 21 days, starting from cycle 2, day 1 (C2D1).

This study was conducted according to the guidelines of the Declaration of Helsinki and was approved by the Ethics Committee in Bern, Switzerland (decision number: 2021-01294; date of approval: 9 December 2021).

### 2.2. Study Endpoints

The primary endpoint of this study was clinical outcome after glofitamab treatment in DLBCL patients according to germline variants *LAG3* rs870849 and *CTLA4* rs231775. The secondary endpoints of this study were toxicities after glofitamab treatment, including cytokine release syndrome (CRS) and immune-effector cell-associated neurotoxicity syndrome (ICANS).

### 2.3. Statistical Analysis

Progression-free survival (PFS) was defined as the time interval from cycle 1 day 1 (C1D1) to progression, relapse, death, or cut-off date 1 November 2025. Overall survival (OS) was defined as the time interval from C1D1 to death from any cause or cut-off date 1 November 2025. Baseline characteristics were analyzed according to *LAG3* rs870849 and *CTLA4* rs231775 somatic genetic variants. Continuous variables are reported as median and interquartile range (IQR), while categorical variables are presented as counts and percentages. Comparisons of continuous variables between groups were performed using the Kruskal–Wallis test. Categorical variables were compared using the Chi-square test or Fisher’s exact test when expected cell counts were <5. Survival outcomes were estimated using Kaplan–Meier curves and compared between genetic groups using the log-rank test. In addition, univariable and multivariable Cox proportional hazards regression models were fitted to estimate the hazard ratios and corresponding 95% confidence intervals. Multivariable models were adjusted for age, sex, prior CAR T-cell therapy, prior high-dose chemotherapy with autologous stem cell transplantation (HDCT/ASCT), number of prior lines of therapy, and remission status before glofitamab treatment. All analyses were done in R Version 4.5.1.

### 2.4. LAG3 and CTLA4 Gene Analysis

Peripheral blood monocytes (PBMCs) were collected prior to glofitamab treatment. Genomic DNA was extracted; *LAG3* gene exon seven and *CTLA4* gene exon one were amplified and sequenced as previously described [15,16].

## 3. Results

### 3.1. Baseline and Clinical Characteristics

This retrospective observational study included 28 R/R DLBCL patients treated with glofitamab at the University Hospital of Berne, Switzerland. Most patients were multi-drug resistant and refractory to CAR-T-cell therapy. The patient characteristics were analyzed in the entire cohort and in the three rs870849 genetic subgroups *LAG3* T455hom, I455Thet, and I455hom (Table 1). The median age at initial diagnosis ranged from 62 years in the I455Thet and I455hom subgroups to 72 years in the T455hom subgroup (*p* = 0.16). In the *LAG3* genetic subgroups, males and females were distributed unequally, with male predominance in the T455hom subgroup and female predominance in the I455hom subgroup (*p* = 0.0001). The majority of patients presented with Ann Arbor stage IV. The T455hom and I455Thet subgroups incorporated a higher proportion of de novo DLBCL (57 and 73%), with equal proportions of de novo and transformed DLBCL in the I455hom subgroup. All patients received R-CHOP as first-line immuno-chemotherapy, and most patients received radiotherapy prior to glofitamab therapy. In total, 25% of the patients had a pretreatment with HDCT/ASCT and 75% had a prior CAR T-cell therapy, with a significantly lower proportion of patients receiving CAR T-cell therapy in the I455hom subgroup (*p* = 0.029).

The patient characteristics were separately analyzed in the three rs231775 genetic subgroups *CTLA4* A17hom, T17Ahet, and T17hom (Table 2). The median age at initial diagnosis ranged from 62 years in the T17Ahet subgroup to 69–70 years in the T17hom and A17hom subgroups, respectively (*p* = 0.56). In the *CTLA4* genetic subgroups, both sexes were equally represented. The T17Ahet and T17hom subgroups had higher proportions of de novo DLCBL (64% and 73%, respectively) compared to the A17hom subgroup (33%). Most patients in the T17hom subgroup received radiotherapy, and the majority of patients across all genetic subgroups received CAR T-cell therapy prior to glofitamab treatment.

### 3.2. Clinical Outcomes After Glofitamab Therapy

**Figure 1:**
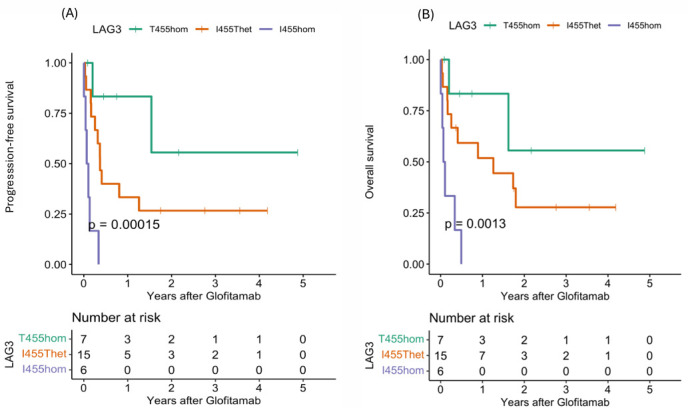
Clinical outcomes in R/R DLBCL patients with glofitamab treatment according to *LAG3* rs870849 genotype. Clinical outcomes in the three genetic subgroups *LAG3* T455hom (green), I455Thet (orange) and I455hom (lilac) were analyzed for (**A**) progression-free survival (PFS) and (**B**) overall-survival (OS).

**Figure 2:**
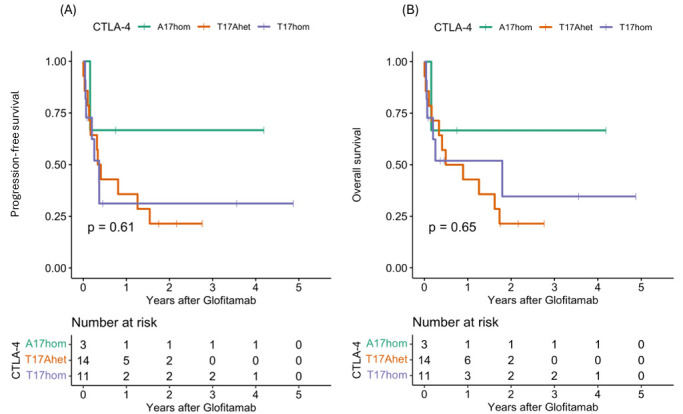
Clinical outcomes in R/R DLBCL patients with glofitamab treatment according to *CTLA4* rs231775 genotype. Clinical outcomes in the three *CTLA4* genetic subgroups CTLA A17hom (green), T17Ahet (orange) and T17hom (lilac) were analyzed for (**A**) progression-free survival (PFS) and (**B**) overall-survival (OS).

The clinical outcomes were analyzed in the entire cohort and in the three *LAG3* genetic subgroups (Table 3). In the I455hom subgroup, 50% of the patients developed CRS following glofitamab treatment, with one patient each experiencing grade 2, 3 and 4 CRS. In contrast, fewer than one third of patients in the I455Thet and T455hom subgroups developed CRS after glofitamab treatment. Two patients experienced grade 2 ICANS following glofitamab treatment: one in the T455hom and one in the I455hom subgroup. One third of the patients experienced relapse, with a higher proportion in the I455hom subgroup (50%). The complete remission (CR) rate was high in the T455hom and I455het subgroups (40–50%), with no overall response in the I455hom subgroup (*p* = 0.07). Dose-dependent effects of LAG3 rs870849 emerged in the analysis of progression-free survival (PFS) and overall survival (OS) in the three *LAG3* genetic subgroups: T455hom, I455Thet, and I455hom (Figure 1A,B). While the T455hom genetic variant was associated with favorable outcomes with median PFS of 19 months and one-year PFS and OS rates of 83%, the I455Thet genetic variant was associated with inferior outcomes with median PFS of 4 months and one-year PFS and OS rates of 31% and 45% and the I455hom genetic variant was associated with dismal outcomes with early disease progression and death (PFS *p* = 0.0002, OS *p* = 0.013, PFz and OS rates *p* = 0.0001). In fact, all of the I455hom patients had progressive disease as the best response, indicating resistance to glofitamab therapy. In the multivariate analysis, *LAG3* I455hom was an indicator of inferor treatment outcome with a hazard ratio (HR) of 14 in PFS (*p* = 0.002) and a HR of 16 in OS (*p* = 0.004) compared to T455hom (Table 4). The number of prior therapy lines was associated with inferior treatment outcome with a HR of 2.6 in PFS (*p* = 0.022) and a HR of 3.1 in OS (*p* = 0.007). Surprisingly, patients with a CAR T-cell therapy prior to glofitamab treatment had a superior response to glofitamab with HR 0.2 in PFS and OS (*p* = 0.08). No significant differences were detected in the three *CTLA4* genetic subgroups in respect to clinical outcomes (Table 5, Figure 2). CRS and ICANS, however, were absent in the A17hom and prevalent in the T17hom subgroup, indicating an association of *CTLA4* genotype with T-cell activity.

## 4. Discussion

In this retrospective observational study, we evaluated treatment outcomes in patients with relapsed/refractory DLBCL after glofitamab treatment, stratified according to the *LAG3* rs870849 genetic variants T455hom, I455Thet, and I455hom, and separately according to *CTLA4* rs231775 genetic variants. Clinical outcome to glofitamab therapy was associated with *LAG3* genotype of the recipients. PFS and OS analyses demonstrated inferior outcomes in *LAG3* I455hom, superior outcomes in *LAG3* T455hom, and intermediate outcomes in *LAG3* I455Thet, a response pattern consistent with the dose-dependent effects of *LAG3* rs870849 in DLBCL treated with glofitamab. In contrast, clinical outcomes of glofitamab therapy were not associated with the *CTLA4* genotype of the recipients. In a previous study in DLBCL treated with CD19-directed CAR T-cell therapy, *LAG3* rs870849 had dominant positive effects on survival and was not dose-dependent, while *CTLA4* rs231775 had dose-dependent effects on survival [15,16]. Therefore, it can be assumed that LAG3 and CTLA4 functions may differ in anti-CD19 CAR T-cell therapy and anti-CD20 bsAB therapy.

The LAG3 polymorphism may regulate T-cell function by altering the inhibitory signaling capacity, thereby modulating T-cell activation, proliferation, and differentiation. LAG3 receptor localizes to the immunological synapse and associates with the T-cell receptor (TCR)-CD3 complex in CD4+ and CD8+ T-cells [24,25,26]. Variants that reduce LAG3 expression result in lower inhibitory signaling at the immunological synapse, leading to enhanced T-cell activation and increased proliferation [27]. Polymorphisms altering LAG3 expression or function may influence the threshold for T-cell activation, given that LAG3 suppresses T-cell activation by disrupting TCR-CD3 signaling [24]. In allogenic hematopoietic stem cell transplantation, LAG3 polymorphism in donor cells can enhance T-cell reactivity and lead to severe Graft versus host disease (GVHD) [27,28]. The rs870849 snp causes a substitution of isoleucine to threonine (I455T) within the LAG3 transmembrane domain, affecting protein processing and proteolytic cleavage [28]. In the R/R DLBCL LAG3 I455hom carriers, glofitamab treatment triggered an abortive T-cell activation accompanied by excessive inflammatory cytokine production, but did not induce the sustained cytolytic activity necessary for tumor destruction, due to a combination of low efficacy and high toxicity, characteristic of an abnormal immune response. This biochemical disorder may result from decreased cleavage of the LAG3 molecule at the cell surface, leading to T-cell dysfunction.

This is the first study that investigates the impact of *LAG3* and *CTLA4* polymorphisms on treatment outcomes in relapsed, refractory DLBCL patients treated with glofitamab. Efficacy, safety, and clinical endpoints after glofitamab treatment have been previously studied [5,11]. Other studies revealed that *LAG3* and *PD1* gene expression were significantly upregulated in DLBCL [19,20,29]. Lee et al. investigated the expression patterns of immune checkpoint receptors in DLBCL and revealed a unique LAG3 expression pattern distinct from other immune checkpoint receptors in DLBCL and proposed a combination therapy of LAG3- and PD-1-inhibitors in the treatment of DLBCL [30].

This study has several limitations. The retrospective single-center study design and the small sample size with only a few patients in the genetic subgroups may have introduced selection bias and affected the generalizability. Due to the small sample size, this investigation is explorative, including the risk of false positive and false negative results. Furthermore, due to the lack of a control cohort, it was not possible to differentiate between prognostic and predictive value. Certain imbalances in the baseline characteristics, particularly the different prior treatments with CAR T-cell therapy, may have also influenced the outcomes. Only 33% of patients in the I455hom subgroup received CAR-T-cell therapy, whereas more than 85% of patients in the T455hom and the I455het subgroup were treated with CAR-T-cell therapy before glofitamab treatment. Also, 83% of the I455hom subgroup were men, whereas in the other genetic subgroups, men and women were equally represented, indicating an imbalance that may have influenced the results. Furthermore, as most patients were not only diagnosed with R/R DLBCL but were also multi-drug resistant and refractory to CAR-T therapy, the results of this study cannot be generalized to R/R DLBCL. Objective parameters were assessed and a multivariate analysis was performed to evaluate the potential influence of confounding factors on the observed outcomes. The multivariate analysis identified the I455hom genotype as the most significant predictor of both PFS and OS, suggesting that the genetic characteristics of the inhibitory cell surface receptor may have greater prognostic relevance than established clinical factors such as sex, age, or number of prior therapies. The use of germline SNPs as risk factors may enable the early identification of ‘high-risk’ R/R DLBCL patients, for example, those with the LAG3 I455H homozygous genotype. For these ‘high-risk’ patients, combination therapy with glofitamab may prevent early disease progression and improve survival outcomes.

## 5. Conclusions

This retrospective single-center study reveals a significant association between *LAG3* rs870849 genotype and treatment outcomes in R/R DLBCL treated with glofitamab, with inferior outcomes in carriers of the *LAG3* I455 genetic variant. The *LAG3* SNP rs870849 may serve as a prognostic biomarker for treatment response in patients with R/R DLBCL receiving glofitamab treatment. To validate this hypothesis, further investigations and larger, prospective studies are required. Mechanistic studies will be required to analyze the projected differential functions of the LAG3 variant proteins.

## Figures and Tables

**Table 1:** Baseline and clinical characteristics according to *LAG3* rs870849 genotype.

	All Patients	T455hom	I455Thet	I455hom	*p*
Number, *n* (%)	28 (100)	7 (25)	15 (54)	6 (21)	
Age at ID (y) median (range)	63 (33, 78)	72 (57, 78)	62 (33, 77)	62 (57, 67)	0.16
Male sex, *n* (%)	15 (54)	3 (43)	7 (47)	5 (83)	0.25
Sex ratio (m/f)	1.15	0.75	0.88	5.0	0.0001
Initial Ann Arbor stage, *n* (%)					0.59
II	3 (11)	2 (29)	0	1 (17)	
III	3 (11)	1 (14)	2 (13)	0	
IV	16 (56)	3 (43)	10 (67)	3 (50)	
nd	6 (22)	1 (14)	3 (20)	2 (33)	
DLBCL					0.58
de novo, *n* (%)	18 (64)	4 (57)	11 (73)	3 (50)	
transformed, *n* (%)	10 (36)	3 (43)	4 (27)	3 (50)	
Prior treatment lines					
n, median (range)	2.5 (2, 3)	2 (2, 3)	3 (2, 3)	2.5 (2, 3)	0.85
Radiotherapy, *n* (%)	17 (71)	5 (71)	8 (53)	4 (67)	0.68
HDCT/ASCT, *n* (%)	7 (25)	1 (14.3)	5 (33)	1 (17)	0.55
CART, *n* (%)	21 (75)	6 (86)	13 (87)	2 (33)	0.029
Age at glofitamab therapy (years) median (range)	66 (35, 80)	73 (59, 80)	65 (35, 78)	67 (58, 73)	0.23

ID: initial diagnosis; DLBCL: diffuse large B-cell lymphoma; HDCT: high-dose chemotherapy; ASCT: autologous stem cell transplantation, nd: not determined; CART: CAR T-cell therapy; m: male; f: female; *n*: number.

**Table 2:** Baseline and clinical characteristics according to *CTLA4* rs231775 genotype.

	All Patients	A17hom	T17Ahet	T17hom	*p*
Number, *n* (%)	28 (100)	3 (10)	14 (50)	11 (40)	
Age at ID (y) median (range)	63 (33, 78)	70 (53, 74)	62 (46, 77)	69 (33, 78)	0.56
Male sex, *n* (%)	14 (50)	1 (33)	8 (57)	6 (55)	0.75
Sex ratio (m/f)	1	0.5	1.33	1.2	0.87
Initial Ann Arbor stage, *n* (%)					0.13
II	3 (11)	0 (0)	3 (21)	0 (0)	
III	3 (11)	0 (0)	2 (14)	1 (9)	
IV	16 (57)	3 (100)	4 (29)	9 (81)	
nd	6 (21)	0 (0)	5 (36)	1 (9)	
DLBCL					
de novo, *n* (%)	18 (64)	1 (33)	9 (64)	8 (73)	0.45
transformed, *n* (%)	10 (36)	2 (66)	5 (36)	3 (27)	
Prior treatment lines					
n, median (range)	8.5 (30)	3 (2.5, 3.5)	2.5 (2, 3)	3 (2, 3)	0.33
Radiotherapy, *n* (%)	17 (61)	1 (33)	7 (50)	9 (82)	0.16
HDCT/ASCT, n (%)	7 (25)	1 (33)	5 (36)	1 (9)	0.29
CART, *n* (%)	21 (75)	2 (67)	10 (71)	9 (82)	0.79
Age at Glofitamab therapy (years) median (range)	66 (35, 80)	72 (61, 77)	65 (59, 78)	70 (46, 80)	0.56

ID: initial diagnosis; DLBCL: diffuse large B-cell lymphoma; HDCT: high-dose chemotherapy; ASCT: autologous stem cell transplantation; CART: CAR T-cell therapy; nd: not determined; m: male; f: female; *n*: number.

**Table 3:** Outcome in Glofitamab therapy according to *LAG3* rs870849 genotype.

	All Patients	T455hom	I455Thet	I455hom	*p*
Number, *n* (%)	28 (100)	7 (25)	15 (54)	6 (21)	
CRS, *n* (%)	9 (32)	2 (29)	4 (27)	3 (50)	0.57
Grade 1	2 (7)	1 (14)	1 (7)	0 (0)	
Grade 2	4 (14)	1 (14)	2 (14)	1 (17)	
Grade 3	1 (4)	0	0	1 (17)	
Grade 4	2 (7)	0	1 (7)	1 (17)	
ICANS, *n* (%)	2 (7)	1 (14)	0	1 (17)	0.29
Relapse, *n* (%)	9 (32)	2 (28)	4 (27)	3 (50)	0.57
Best Response					
CR	9 (32)	3 (50)	6 (40)	0	0.07
PR	2 (7)	1 (17)	1 (7)	0	
SD	2 (7)	1 (17)	1 (7)	0	
PD	14 (50)	1 (17)	7 (47)	6 (100)	
PFS (mo) median	4	19	4	1	0.0002
OS median	6	>48	11	1	0.0013
1-year PFS rate (%)	35	83	31	0	0.0001
1-year OS rate (%)	43	83	45	0	0.0001

CRS: cytokine release syndrome; ICANS: immune effector cell-associated neurotoxicity syndrome; CR: complete remission; PR: partial remission; SD: stable disease; PD: progressive disease; PFS: progression-free survival; OS: overall survival.

**Table 4:** Clinical outcome hazard ratios (HRs); multivariate analysis.

	OS			PFS		
Characteristics	HR	95% CI	*p*	HR	95% CI	*p*
*LAG3* I455Thet vs. T455hom	3.97	0.79, 20.1	0.10	3.53	0.63, 19.6	0.15
*LAG3* I455hom vs. T455hom	16.4	2.75, 97.4	0.002	14.2	2.31, 87.0	0.004
Age	0.94	0.32, 2.80	>0.9	1.41	0.48, 4.21	0.5
Male sex	0.89	0.30, 2.63	0.8	0.86	0.29, 2.55	0.8
CAR T-cell therapy prior to glofitamab	0.22	0.04, 1.19	0.079	0.21	0.04, 1.19	0.08
Number of prior therapy lines	2.62	1.15, 6.01	0.022	3.11	1.37, 7.05	0.007

HR: hazard ratio; CI: Confidence interval; OS: overall survival; PFS: progression-free survival.

**Table 5:** Outcome in Glofitamab therapy according to *CTLA4* rs231775 genotype.

	All Patients	A17hom	T17Ahet	T17hom	*p*
Number, *n* (%)	28 (100)	3 (11)	14 (50)	11 (39)	
CRS, *n* (%)	9 (32)	0	3 (21)	6 (55)	0.12
Grade 1	2 (7)		0	2 (33)	
Grade 2	4 (14)		1 (33)	3 (50)	
Grade 3	1 (4)		1 (33)	0	
Grade 4	2 (7)		1 (33)	1 (17)	
ICANS, *n* (%)	2 (7)	0	0	2 (18)	0.19
Relapse, *n* (%)	9 (32)	0	5 (36)	4 (36)	0.45
Best Response					0.35
CR	9 (32)	2 (67)	4 (29)	3 (30)	
PD	14 (50)	1 (33)	8 (57)	5 (50)	
PR	2 (7)	0	2 (14)	0	
SD	2 (7)	0	0	2 (20)	
PFS median	4	>48	4	5	0.61
OS median	6	>48	6	22	0.65

CRS: cytokine release syndrome; ICANS: immune effector cell-associated neurotoxicity syndrome; CR: complete remission; PD: progressive disease; PR: partial remission; SD: stable disease; PFS: progression-free survival; OS: overall survival; *n*: number.

## Data Availability

All primary data can be provided upon request.
